# Effects of administration of sildenafil citrate and amino acids on selected physiological, blood gas and biochemical parameters in pregnant twin-bearing sheep in late gestation

**DOI:** 10.1016/j.vas.2026.100664

**Published:** 2026-04-17

**Authors:** Leesa J Dunstan, Sue A McCoard, Michelle L Hebart, Forbes D Brien, Colin L Trengove, Mariana Caetano

**Affiliations:** a Davies Livestock Research Centre, School of Animal and Veterinary Sciences, Adelaide University, Roseworthy, South Australia 5371, Australia; b AgResearch Group, Bioeconomy Science Institute, Palmerston North 4472, New Zealand

**Keywords:** Blood gases, Multiparous pregnancy, Nitric oxide, Sheep, Viagra

## Abstract

Sildenafil citrate (SC) and specific amino acids such as arginine have been shown to increase fetal oxygen availability when administered individually to pregnant ewes, however, the effects of both compounds in combination are not well known. It is therefore important to understand the physiological effects of combining SC with amino acids (AA) in the pregnant ewe when administered during pregnancy. This preliminary study, which was designed as a safety trial, investigated physiological, blood gas and biochemical parameters in twin-bearing ewes when SC and arginine were supplemented in an AA blend. During late gestation, 32 twin-bearing Merino ewes were allocated to one of four groups: control, AA blend, SC and AA blend + SC (AA + SC). The AA blend was intravenously administered over three infusion periods, daily, to the AA and AA + SC groups. The SC and AA + SC groups were given SC as a subcutaneous injection (150 mg/d), also split over three time periods. Reverse osmosis water and saline were used as controls within treatments. Blood ketone and glucose levels were monitored daily, and heart rate (bpm), respiration rate (bpm), rectal temperature ( °C) and venous blood gases were measured at 0, 2, 6 and 10 d of treatment. Average parameters did not measure outside of normal reported ranges for sheep and no adverse effects were observed, indicating the safety of arginine in an AA blend in combination with sildenafil citrate to twin bearing ewes. It is important to be aware of the potential effects of administration of arginine and sildenafil citrate combined, in various dosage rates, for future studies.

## Highlights

• Sildenafil + arginine caused no adverse effects on physiology or blood gases in late-gestation twin-bearing ewes.
• 150 mg/day sildenafil citrate did not increase blood oxygen in pregnant ewes.
• This novel study adds to knowledge of compounds used in IUGR research in sheep models.

## Introduction

Pregnancy preserves the genetic integrity of a species by sustaining its reproductive capacity, changes in hormones, cytokines, enzymes, and nutrition are all crucial components of this process (Ajafar et al., 2022; Al-Jaryan et al., 2023). Maternal nutrition during gestation, especially dietary protein intake, is a key determinant in embryonic survival, growth, and development (Herring et al., 2018). Recent studies investigating nutritional strategies to mitigate the consequences of increased nutritional demand have explored the role of specific amino acids (AA), rather than protein as a macronutrient (Cao et al., 2021). Many AA play significant roles in the development and overall programming of a fetus during gestation, when maternally supplemented (Hussain et al., 2020; McCoard et al., 2016). The major challenge when attempting to supplement the maternal circulation for intentional impact on the fetal circulation is ensuring adequate utero-placental blood flow (Lang et al., 2003). Uteroplacental blood flow increases substantially during the last stages of pregnancy as the fetus is primarily growing rather than developing (Wareing et al., 2005). As well as influencing blood flow, oxygen supply also contributes directly to the metabolism and transport of AA within the feto-placental unit (Duggleby & Jackson, 2002; Kramer et al., 2023).

Compounds that increase oxygen availability to a growing fetus when administered to pregnant ewes include sildenafil citrate (SC) (Satterfield et al., 2010) and specific amino acids including arginine (Satterfield et al., 2013), leucine (Yang et al., 2015) and glutamine (Durante, 2019). Sildenafil citrate stimulates vasodilation by delaying the breakdown of cyclin guanosine monophosphate (cGMP) and has been observed to rapidly reduce mean arterial pressure and output while simultaneously increasing the heart rate and blood flow to the uterus in ovine models (Dishy et al., 2001; Wareing et al., 2005). The messenger molecule cGMP is responsible for the regulation of downstream effects on vasodilation, as well as retinal phototransduction, calcium homeostasis and neurotransmission (Zbrojkiewicz & Śliwiński, 2016). Arginine also promotes vasodilation by increasing concentrations of nitric oxide (NO) in the blood (Maul et al., 2003; Satterfield et al., 2013). As a product of arginine catabolism, NO regulates the growth of new blood vessels in existing vasculature and thus influences utero-placental blood flow (Reynolds & Redmer, 2001; Satterfield et al., 2013).

The use of both SC and arginine, individually, have been tested in ovine models without adverse effects (Satterfield et al., 2010; Satterfield et al., 2013; McCoard et al., 2013; Peterson et al., 2016), but the effects of administration of both compounds, in addition to leucine and glutamine, in combination have not been explored. Although these compounds stimulate vasodilation by different pathways, both lead to an increase in blood flow. While increased blood flow may be beneficial during pregnancy, overstimulation of NO production can be detrimental (Petros et al., 1991). For example, increased concentrations of NO can cause excess creatine and urea in the blood stream (Ho et al., 2003), adding strain to already highly functioning kidney (Lopes Van Balen et al., 2019) and potentially lead to systematic hypotension, or high blood pressure (Umans & Levi, 1995).

As a safety study, the aim of this preliminary trial was to evaluate the potential for adverse effects of administration of a combination of SC, arginine, leucine and glutamine (in an AA blend), on selected physical (e.g. live weight and feed intake), and physiological parameters (e.g. blood chemistry and gases) ewes in late pregnancy, prior to a larger-scale trial to evaluate potential animal health and productivity impacts. The overall AA blend included Glu, Gln, Arg, His, and Leu due to their roles in fetal growth and development, with doses based on the variation seen between single and twin fetuses by Van der Linden et al., 2013. It was hypothesised that there would be no adverse effects of administration of a combination of SC and selected AA on ewe physical or physiological parameters.

## Materials and methods

This trial was conducted in accordance with the guidelines provided by the Australian Code for the Care and Use of Animals for Scientific Purposes (National Health & Medical Research Council, 2013). The use of animals was approved by Adelaide University Animal Ethics Committee (S-2020-061). Experimental work was conducted at the Adelaide University, Roseworthy Campus, Roseworthy, South Australia, Australia.

### Experimental design

#### Animals and experimental design

Multiparous Merino ewes (excluding maidens) were synchronised with progesterone controlled intrauterine drug release devices (CIDRs). Following removal of the CIDRs, ewes were mated naturally over one oestrus cycle (21 d). Thirty-two twin bearing ewes were identified via transabdominal ultrasonography and managed under commercial grazing conditions until entering the indoor animal house at 125 d of gestation.

From 125 d to 135 d of gestation, the ewes were moved to the animal house facility for general acclimatisation to individual pens (1 m^2^), a total mixed ration (TMR) diet and daily handling. During the 10 d acclimatisation period heart rate (bpm; Littmann Stethoscope (Multipoint Technologies Pty Ltd., Dandenong, Victoria, Australia), respiratory rate (bpm; manual observation) and rectal temperature ( °C; Welcare, Fishermans Bend, Victoria, Australia) were recorded for each ewe. On 129 d of gestation all ewes were fitted with intravenous jugular catheters (16 G 3.25″ 2.1 × 83 mm Angiocath catheter with 30 cm microbore T-connector, luer lock, extension set, Provet Australia, South Australia, Australia) for AA and control saline administration and blood sample collection.

Ewes (*n* = 8 per group) were allocated into four treatment groups: 1) AA blend and SC (AA+SC), 2) AA and reverse osmosis (RO) water injection, 3) SC and saline infusion, and 4) control (saline infusion and RO water injection), via stratified randomization for age (4.5 *y* ± 0.8), live weight on 125 d of pregnancy (78 kg ± 9.5) and body condition score on 125 d of pregnancy (3.7 ± 0.4; 1–5 scale, (Jefferies, 1961). Saline infusions and RO water injections were given to replicate the same interventions performed for other treatment groups delivering AA and SC, respectively. Control saline and AA were administered via the jugular catheter. Injections (RO water and SC) were administered on alternating sites on the ewe’s rump. Treatment began at 135 d of gestation. Treatments were administered thrice daily (06:00, 14:00 and 22:00). Between treatments, catheters were flushed with heparinised saline solution (0.9 % sodium chloride, Baxters Healthcare Pty Ltd, Old Toongabbie, Australia: 10 U/mL sodium heparin) to maintain patency.

The AA blend solution was prepared using 19.7 g of L-Arginine, 18.2 g of L-Histidine, 7.8 g of L-Leucine, 22.9 g of L-Glutamine and 11.4 g of L-Glutamic acid (all sourced from Sigma Aldrich Pty Ltd, St Louis, Missouri USA), and dissolved in 960 mL of sterilised RO water at 600 rpm at 25 °C. Sterilisation was performed by autoclaving 2 L volumes of RO water at 120–135 °C prior to AA being added. Each individual animal AA blend, single-dose, infusion consisted of 0.4 g of L-Arginine, 0.37 g of L-Histidine, 0.17 g of L-Leucine, 0.47 g of L-Glutamine and 0.23 g of L-Glutamic acid in a 20 mL saline bolus thrice daily. These specific amounts of AA were calculated to provide the difference in AA plasma concentrations between full term single and twin fetuses identified by Van der Linden et al., 2013. The SC (Pfizer, New York, NY) was prepared by dissolving 2.4 g of SC in 432 mL of sterilised reverse osmosis (RO) water on a stirrer at 600 rpm at 25 °C. The SC was administered after the AA infusions, where 50 mg of SC was administered in a 9 mL RO water bolus. Dosage of SC was determined as the highest concentration previously given to multiparous ewes to produce positive effects on uterine blood flow during pregnancy (Satterfield et al., 2010). Both the AA blend and SC were prepared no more than two days in advance, in bulk, for daily administration. Solutions were stored at 4 °C, away from direct light. Individually aliquoted doses were removed from storage to warm to room temperature for 10 min before administration.

#### Nutrition

A total mixed ration was provided to meet 100 % of National Research Council requirements for twin bearing ewes, delivering 2.5 % of bodyweight of DM, based on individual body weights at entering the animal house at 125 d of gestation ((National Research Council, 2007); Table 1, Table 2). Ewes were fed once daily at 09:00 and any daily refusals were recorded the following day for the duration of the trial period. Any remaining feed was dried in a fan-forced oven at 55 °C to determine dry matter for measurement of individual intakes. Average intakes over the supplementation period were 0.83 kg ± 0.44 for AA+SC, 0.97 kg ± 0.38 for AA, 1.11 kg ± 0.33 for SC and 1.03 kg ± 0.32 for the control group.

**Table 1:** Composition of concentrate (on a DM basis).

Item	g/kg
Oats	39.05
Barley	38.92
Lupins	17.35
Buffer	1.50
Vegetable oil	1.08
Urea	0.64
Calciprill, 36 % Ca	0.55
Salt	0.22
Mineral & vitamin mix	0.22
Vitamin E, 5 %	0.43
Bovatec, 20 %	0.04

Manufactured by Hills Farm Supplies, Mt Barker, South Australia, Australia. Nutritional specifications provided via Near-Infrared Spectroscopy (AgriNIR, Dinamica Generale, Poggio Rusco, Italy).

**Table 2:** Nutritional composition of the total mixed ration feed offered to pregnant ewes per kg of dry matter.

Nutrients	Analysis
Dry matter ( %)	100
ME (MJ/kg)	10.94
Crude protein (g/kg)	0.15
NDF (g/kg)	0.36
Fat (g/kg)	0.03
Calcium (g/kg)	0.008
Phosphorus (g/kg)	0.003
Magnesium (g/kg)	0.002
Potassium (g/kg)	0.014
Sulphur (g/kg)	0.002
Sodium (g/kg)	0.0007
Chloride (g/kg)	0.004
Zinc (mg/kg)	60.00
Cobalt (mg/kg)	0.50
Copper (mg/kg)	0.00
Iodine (mg/kg)	0.50
Manganese (mg/kg)	20.00
Selenium (mg/kg)	0.10
Vitamin A kiu/kg	4.50
Vitamin D kiu/kg	0.50
Vitamin E kiu/kg	40.00
Vitamin B1 (mg/kg)	1000.00
Bovatec (mg/kg)	33.00

#### Sampling

Baseline rectal temperature ( °C), heart rate (bpm), respiratory rate (bpm), ketones (mmol/L; LS-946; Genesis Biotech Pty Ltd, Borkstraβe, Münster, Germany) and blood gas parameters using i-Stat Alinity point-of-care analyser and CG8+ cartridges (Abbott Point of Care, i-Stat Alinity, Abbott Park, Illinois, USA), were recorded for each ewe on 129 d of gestation. Respiratory rate was taken by observing and counting the inspiration (bpm) prior to any handling or other baseline measurements to avoid elevation due to stress. Rectal temperature, heart rate, respiratory rate and all blood parameters and gases were collected at 14:00 h prior to treatment administration on 2, 6 and 10 d post commencement of treatment corresponding to approximately 137, 141, and 144 d of gestation respectively.

Blood was collected via the fitted intravenous jugular catheters. Whole blood collected on 137, 141 and 144 d was analysed for blood gas concentrations immediately following collection to measure blood pH, partial pressure of carbon dioxide (pCO_2_), partial pressure of oxygen (pO_2_), bicarbonate (HCO_3_), base excess (BE), saturated oxygen (SO_2_), total carbon dioxide (TCO_2_), sodium (Na), potassium (K), calcium (Ca), glucose (mmol/L), haematocrit (L/L) and haemoglobin (Hgb).

#### Statistical analysis

Ewe data were analysed using a Linear Mixed model in SPSS (IBM SPSS, version 27, Armonk, NY, USA). The final model included treatment (AA+SC vs AA vs SC vs control), day (0, 2, 6, and 10) and the interaction between treatment and day as fixed factors. Model residuals were assessed for normality using Q-Q plots in SPSS. Residuals showed no major deviations from normality. Residual vs predicted plots of standardized residuals for each variable indicated homogeneous variance. No evidence of heteroscedasticity was observed. Ewe ID was fitted as a random effect with an unstructured covariance structure to account for repeated measures, allowing for individual-specific intercept and accommodating inherent between-ewe differences and non-independence of repeated observations over time. The interaction between treatment and sampling day has not been reported as it was not statistically significant for any variable. Probabilities <0.05 were considered statistically significant, while values >0.05 and <0.10 were considered a trend.

## Results

Treatment had no effect on ewe rectal temperature, heart rate, or respiratory rate measured over the trial period (Table 3). Sampling day had a small effect on rectal temperature, but heart rate and respiratory rate were unaffected by sampling day, and no treatment by sampling day interactions were evident. There was no adverse health events related to the treatment during the trial period.

**Table 3:** Ewe rectal temperature, heart rate, and respiratory rate values (mean ± SE) and probabilities for each treatment and sampling day.

	Treatment					Day				
	AA+SC	AA	SC	Control	*P-value*	0	2	6	10	*P-value*
Rectal temperature ( °C)	39.0 ± 0.12	38.9 ± 0.12	39.1 ± 0.12	39.0 ± 0.12	0.96	38.6^a^ ± 0.10	39.3^b^ ± 0.10	38.9^c^ ± 0.10	39.3^b^ ± 0.13	<0.001
Heart rate (bpm)	74.2 ± 4.21	74.1 ± 4.21	77.9 ± 4.21	81.5 ± 4.21	0.56	74.4 ± 3.87	82.3 ± 3.87	79.6 ± 3.87	71.5 ± 4.91	0.27
Respiratory rate (bpm)	53.7 ± 5.42	65.4 ± 5.42	54.2 ± 5.42	59.5 ± 5.42	0.35	54.5 ± 3.70	59.8 ± 3.70	62.4 ± 3.70	56.0 ± 4.49	0.26

0 = baseline; 2 = two days of treatment; 6 = six days of treatment; 10 = ten days of treatment.

AA+SC = amino acid and blend sildenafil citrate; AA = amino acid blend and RO water; SC = sildenafil citrate and saline; Control = saline and RO water.

^a,b,c^ Significant differences in means are represented by different letters in the same row within each classification.

Treatment had an effect on PO_2_, where the control group had lower PO_2_ than the AA and AA + SC groups but not the SC group (Table 4). A similar trend for a treatment effect was seen in sO_2_ concentrations. There was an effect of sampling day on PCO_2_ where lower PCO_2_ was observed on day 2 and 10 compared to day 0. No other effects of treatment, day, or their interaction were observed for any of the blood gas parameters measured.

**Table 4:** Blood gas values (mean ± SE) and probabilities including pH, partial pressure of carbon dioxide (PCO_2_), partial pressure of oxygen (PO_2_), bicarbonate (HCO_3_), base excess (BE), sulphur dioxide (sO_2_), and total carbon dioxide (TCO_2_) for each treatment and day of blood sampling.

Treatment						Day				
	AA+SC	AA	SC	Control	*P-value*	0	2	6	10	*P-value*
pH	7.46 ± 0.01	7.48 ± 0.01	7.48 ± 0.01	7.48 ± 0.01	0.34	7.47 ± 0.01	7.48 ± 0.01	7.47 ± 0.01	7.48 ± 0.01	0.25
PCO_2_ (mmHg)	34.77 ± 0.89	33.97 ± 0.88	35.68 ± 0.89	36.17 ± 0.89	0.41	35.99^a^ ± 0.51	34.68^b^ ± 0.55	35.16^ab^± 0.53	34.77^b^ ± 0.57	0.02
PO_2_ (mmHg)	46.50^a^ ± 1.36	45.82^a^ ± 1.42	42.33^ab^± 1.41	41.24^b^± 1.41	0.04	43.44 ± 0.95	43.77 ± 1.07	44.41 ± 1.02	44.27 ± 1.55	0.84
HCO_3_ (mmol/L)	24.71 ± 0.86	26.35 ± 0.88	27.07 ± 0.88	26.98 ± 0.88	0.28	26.45 ± 0.52	26.33 ± 0.56	25.89 ± 0.54	26.45 ± 0.59	0.63
BE, ecf (mmol/L)	0.96 ± 0.95	2.98 ± 0.97	3.63 ± 0.97	3.51 ± 0.97	0.25	2.84 ± 0.58	2.96 ± 0.65	2.24 ± 0.62	3.04 ± 0.68	0.63
sO_2_ ( %)	84.49 ± 1.48	84.34 ± 1.55	81.43 ± 1.54	79.24 ± 1.54	0.07	81.76 ± 1.06	82.48 ± 1.19	82.66 ± 1.14	82.58 ± 1.28	0.88
TCO_2_ (mmol/L)	25.73 ± 0.87	27.33 ± 0.89	28.15 ± 0.89	28.15 ± 0.89	0.26	27.55 ± 0.53	27.30 ± 0.57	26.99 ± 0.55	27.54 ± 0.60	0.61

0 = baseline; 2 = two days of treatment; 6 = six days of treatment; 10 = ten days of treatment;.

AA+SC = amino acid and blend sildenafil citrate; AA = amino acid blend and RO water; SC = sildenafil citrate and saline; Control = saline and RO water.

^a,b,c^ Significant differences in means are represented by different letters in the same row within each classification.

Serum biochemical parameters are presented in Table 5. All serum biochemical parameters measured were affected by sampling day. Ketone concentrations were higher on day 6 of sampling, before returning to levels like that of day 0 concentrations at 10 d Potassium, calcium, and Hb Hct were lower on day 0 compared to all other sampling days. Glucose was higher on day 2 and 6 compared to day 0 and 10.

**Table 5:** Serum biochemical values (mean ± SE) and probabilities including blood ketone, sodium, potassium, calcium, glucose, haematocrit (Hct), and haemoglobin (Hb) for each treatment and day.

	Treatment					Day				
	AA+SC	AA	SC	Control	*P-value*	0	2	6	10	*P-value*
Ketone (mmol/L)	1.27 ± 0.10	1.17 ± 0.11	0.94 ± 0.11	1.20 ± 0.10	0.16	1.07^a^ ±0.12	1.30^ab^ ±0.13	1.50^b^ ±0.13	1.10^a^ ±0.15	0.02
Sodium (mmol/L)	149.79 ± 0.32	149.58 ± 0.33	149.33 ± 0.24	149.36 ± 0.33	0.62	149.06^a^ ± 0.22	150.16^b^ ± 0.25	149.33^a^ ± 0.24	150.18^b^ ± 0.26	<0.001
Potassium (mmol/L)	3.86 ± 0.06	3.95 ± 0.06	4.01 ± 0.06	4.10 ± 0.06	0.07	3.87^a^ ± 0.05	3.95^ab^ ± 0.05	4.06^b^ ± 0.05	4.05^b^ ± 0.05	0.02
Calcium (mmol/L)	1.22 ± 0.02	1.19 ± 0.02	1.24 ± 0.02	1.18 ± 0.02	0.12	1.16^a^ ± 0.02	1.21^b^ ± 0.02	1.24^b^ ± 0.02	1.22^b^ ± 0.02	0.01
Glucose (mmol/L)	2.25 ± 0.12	2.15 ± 0.13	2.47 ± 0.13	2.31 ± 0.13	0.40	2.45^a^ ± 0.08	2.24^b^ ± 0.09	2.14^b^ ± 0.08	2.35^a^ ± 0.09	0.01
Hct (L/L)	0.29 ± 0.01	0.29 ± 0.01	0.28 ± 0.01	0.28 ± 0.01	0.54	0.31^a^ ± 0.01	0.28^b^ ± 0.01	0.28^b^ ± 0.01	0.27^b^ ± 0.01	<0.001
Hbˆ (g/L)	100.95 ± 3.14	98.93 ± 3.18	94.36 ± 3.19	96.32 ± 3.19	0.50	104.25^a^ ± 1.80	95.33^b^ ± 1.93	96.75^b^ ± 1.87	94.25^b^ ± 2.01	<0.001

ˆvia Hct 0 = baseline; 2 = two days of treatment; 6 = six days of treatment; 10 = ten days of treatment;.

AA+SC = amino acid and blend sildenafil citrate; AA = amino acid blend and RO water; SC = sildenafil citrate and saline; Control = saline and RO water.

^a,b,c^ Significant differences in means are represented by different letters in the same row within each classification.

## Discussion

The objective of this study was to determine the effect of supplementation of 150 mg/day of SC, a blend of selected AA, a combination of SC and the AA blend compared to a saline control on selected physical and physiological parameters in multiparous twin-bearing ewes in late gestation. Overall, the findings of the study indicated that SC can be safely administered to twin-bearing ewes in late pregnancy in conjunction with an AA blend, with no major adverse effects on selected physical and physiological parameters.

### Effects on physiological parameters

The absence of treatment effects on vital signs of rectal temperature, heart, and respiration rate indicates no adverse effects of the treatments on basic body functions of the late gestation twin-bearing ewe. Rectal temperature was influenced by sampling day, but the range in rectal temperatures (38.6 – 39.3 °C) were within the normal range for rectal temperature (38.5 - 39.5 °C) in sheep (Kearton et al., 2020). These results are in agreement to prior work in which no difference in the maternal heart rate, as a reaction to systematic vasodilation, has been reported between intrauterine growth restricted, mid-gestation singleton-bearing ewes following 100 mg SC bolus and 100 mg 24 hour gradual infusion administration or control (Miller et al., 2009). Average heart rates for each treatment group in the present study were within the reference range of 65–80 bpm (Reece, 2004), except for 2 d where rates were slightly elevated. However, the increase in heart rate was not considered a response to the treatments tested since up to a 50 % increase was expected during handling (Scott, 2015).

Respiratory rate was much higher in the current study than in the noted reference range of 16–34 bpm (Reece et al., 2004), although control respiratory rates of 50 bpm have been recorded in Merinos in previous studies (Johnson, 1991; Srikandakumar et al., 2003). Studies in humans have observed insignificant changes in respiratory rate after taking SC (Snyder et al., 2008; Zusman et al., 2000). Studies in pregnant ewes following arginine supplementation have not reported respiratory rate as a variable measured (Lassala et al., 2010; McCoard et al., 2013; Peine et al., 2018). In the current study, while high, average respiratory rate did not differ between treatment groups or over time. The high respiration rates may be due to high stress during handling due to being housed individually indoors, despite acclimatisation and regular handling.

### Effects on metabolic parameters

For this trial, PO_2_ was used as an indicator of respiratory distress or respiratory acid-base imbalance following administration of SC, AA or their combination. The PO_2_ describes the amount of oxygen that is in the blood at the time of collection and reflects the animal’s respiratory efficiency in oxygen transfer from the surrounding atmosphere to the blood (Reece & Rowe, 2017). The SC + AA group had the highest PO_2_ value compared to other treatment groups, potentially in response to the increased production of the potent vasodilator NO (Kourembanas et al., 1993; Premont et al., 2020). The control and SC group had lower PO_2_ compared to the AA and AA + SC groups, but all PO_2_ values in all animals were similar to levels reported in conscious, untreated sheep (Onmaz et al., 2009; Sobiech et al., 2005).

While not statistically significant, in the current study, blood glucose concentrations were lower in those ewes supplemented with AA (both AA and AA+SC). This decrease in glucose may have occurred through the regulation of serum glucose concentrations by glucokinase, triggering a rise in insulin simultaneously as a product of AA metabolism, and therefore upregulation of glucose deposition in the liver as glycogen (Norton et al., 2022). It should be noted that while sampling day was found to have a statistically significant effect on glucose, levels were within ranges reported elsewhere, although in a smaller sheep breed (2.0 - 4.5 mmol/L; (Gardner et al., 2003). While sampling occurred at the same time each day, the amount of feed and supplement consumed prior to sampling was not monitored, which may cause fluctuations in concentrations that would not be due to the treatments.

Blood calcium was unaffected by treatment but were ∼45 % compared to the lower reference calcium concentrations (2.2–2.7 mmol/L; (Pardi et al., 2004). As the demand for calcium is considerably higher during twin pregnancies compared to singles, particularly during late gestation, it is expected that calcium levels may be low. Low levels may also be attributed to the supply of NO as without the inhibition of cGMP, the activation of cGMP-dependant protein kinase (PKG) leads to a release of intracellular calcium (Wang et al., 2005). In hypoxic rats, SC administration decreases resting calcium concentrations (Pauvert et al., 2004) but reports of low calcium following SC or NO supply has not occurred in other species.

Ketone (β-hydroxybutyric acid; BHBA) levels were measured as an indicator of pregnancy toxaemia during late pregnancy. Ewes in all groups exhibited subclinical ketosis, but there was no effect of treatment on BHBA concentrations. Industry standards define clinical ketosis as blood BHBA concentrations above 1.6 mmol/L, subclinical ketosis as concentrations 0.8 – 1.6 mmol/L, and normal levels below 0.8 mmol/L (Marutsov,a & Marutsov, 2017). Concentrations of all groups in the current study ranged from 0.94 – 1.27 mmol/L. Pregnancy toxaemia is often noticeable through high ketone and low glucose concentrations; however glucose levels were within the lower reference limits in this study. Pregnancy toxaemia is common in multiple bearing ewes in late gestation when glucose demands are high (Schlumbohm & Harmeyer, 2008). The pregnancy status and once daily feeding, rather than ad libitum grazing, may have contributed to the subclinical ketosis observed. It was important to monitor for subclinical pregnancy toxaemia as it can be harmful to the ewe and her lambs by causing a decrease in milk production due to glucose imbalances (McArt et al., 2013) and the increased risk of reproductive disorders and mastitis (Suriyasathaporn et al., 2000).

### Limitations

As previously suggested, the subclinical ketosis observed may have been exacerbated by feeding only once per day. Reducing feeding frequency has been associated with fluctuations in rumen pH and metabolic load (Chen et al., 2024), potentially increasing the reliance on lipid metabolism and ketone production (Mao & Wang, 2025; Simões & Margatho, 2024). The frequency of feeding should be addressed for future studies to ensure more stable energy supply to reduce the risk of subclinical ketosis and confounding treatment effects.

It is also important to note that the current study did not investigate fetal endpoints, such as birth weight, viability of neonatal metabolic parameters. Research suggests that maternal metabolic imbalances have effects on placental function and fetal development, even at subclinical levels (Corrales-Hlinka et al., 2023; Mitchell et al., 2022). While many studies look at such effects from a maternal undernourishment model, similar effects may have been observed in the offspring in the current study is measured. Assessing outcomes across the meternal-fetal unit would strengthen the understanding of the implications of the treatments investigated.

## Conclusion

Overall, there was no evidence of additional adverse effects on the investigated maternal parameters under the doses and experimental conditions used in late gestation twin-bearing Merinos. All parameters investigated in this study were within known reference ranges for pregnant ewes. These findings indicate that at the levels used in this study, that SC, the AA blend and their combination can be administered to twin-bearing ewes in late gestation without detrimental effects on ewe wellbeing. Future studies should address the limitations of the current research to investigate the effect of treatments on the offspring. Additionally, the feeding regime should be revised to include multiples feeding per day to reduce additional contribution to metabolic stress in multiple bearing ewes. This study is novel and contributes to the knowledge of compounds regularly used in IUGR studies for both sheep and sheep models.

## Data availability

The data supporting this study’s findings are available from the corresponding author upon reasonable request.

## Funding statement

This research did not receive any specific grant from funding agencies in the public, commercial, or not-for-profit sectors.

## Ethical statement

The authors confirm that the *Veterinary and Animal Science* ethical policies have been adhered to, and the appropriate ethical review committee approval has been received for this study involving animals. The use of animals was approved by The University of Adelaide Animal Ethics Committee (S-2020-061). All relevant guidelines for humane animal treatment and welfare have been followed.

## CRediT authorship contribution statement

**Leesa J Dunstan:** Writing – review & editing, Writing – original draft, Visualization, Investigation, Data curation, Conceptualization. **Sue A McCoard:** Writing – review & editing, Validation, Supervision, Methodology, Conceptualization. **Michelle L Hebart:** Writing – review & editing, Visualization, Validation, Supervision, Investigation, Formal analysis, Data curation. **Forbes D Brien:** Writing – review & editing, Visualization, Validation, Supervision, Investigation. **Colin L Trengove:** Writing – review & editing, Investigation. **Mariana Caetano:** Writing – review & editing, Visualization, Validation, Supervision, Methodology, Investigation, Data curation, Conceptualization.

## Declaration of competing interest

The authors declare the following financial interests/personal relationships which may be considered as potential competing interests:

Leesa-Joy Dunstan reports financial support was provided by Alltech Inc. If there are other authors, they declare that they have no known competing financial interests or personal relationships that could have appeared to influence the work reported in this paper.
